# Raising the D-dimer bar: a narrative review of the age-adjusted D-dimer threshold

**DOI:** 10.1016/j.rpth.2025.103255

**Published:** 2025-11-17

**Authors:** Tayssir Fatah, Judith Catella, Christophe Nougier, Hamdi Rezigue

**Affiliations:** 1 Service d'Hématologie Biologique et d’Hémostase clinique, Hospices Civils de Lyon, Bron, France; 2 Service de Médecine Interne et Vasculaire, Hôpital Edouard Herriot, Hospices Civils de Lyon, Lyon, France; 3 Laboratoire Interuniversitaire de Biologie de la Motricité (LIBM) EA7424, Équipe “Biologie Vasculaire et du Globule Rouge,” Université Claude Bernard Lyon 1 Université de Lyon, Lyon, France; 4 UR4609-Hémostase et Thrombose, UFR Laennec, Université Claude Bernard, Lyon, France

**Keywords:** D-dimer, deep vein thrombosis, fibrin, pulmonary embolism, venous thromboembolism

## Abstract

D-dimers play a key role in diagnosing venous thromboembolism (VTE) due to high negative predictive value in excluding VTE in patients with a nonhigh clinical probability. However, D-dimer levels naturally increase with age, complicating their interpretation in elderly patients. To address this, an age-adjusted threshold multiplying the patient’s age by 10 (μg/L), starting from age 50 years, has been proposed in several studies, to exclude the diagnosis of VTE in patients over 50 years with a nonhigh clinical probability. This narrative review discusses the establishment as well as the efficiency and safety of the age-adjusted threshold multiplying the patient’s age by 10 (μg/L), with a focus on the HemosIL D-dimer assays. Overall, the age-adjusted D-dimer threshold has demonstrated enhanced specificity without compromising sensitivity in excluding VTE in patients with suspected pulmonary embolism and nonhigh clinical probability in emergency department settings. By improving specificity, reducing imaging reliance, and lowering costs, the age-adjusted threshold offers a cost-effective and efficient strategy for optimizing VTE management. However, real-world diagnostic strategy studies remain limited, particularly for deep vein thrombosis. Retrospective studies dominate this area, and the cautious stance of scientific societies reflects the absence of large-scale, prospective trials. Emerging evidence suggests the age-adjusted threshold may be as safe and efficient as the conventional approach for deep vein thrombosis exclusion. More than 30 commercial assays are available for D-dimer testing, and the age-adjusted threshold is not validated with all commercially available D-dimer techniques. This underscores the critical need for assay-specific validation before age-adjusted thresholds can be reliably integrated into routine clinical practice.

## Essentials

• D-dimer levels naturally increase with age.
• D-dimers exclude venous thromboembolism with a high negative predictive value.
• An age-adjusted threshold (age × 10 μg/L) has been proposed for patients over 50 years old.
• This threshold is not validated across all commercially available D-dimer assay.

## 1 Introduction

Diagnostic of venous thromboembolism (VTE), encompassing deep vein thrombosis (DVT) and pulmonary embolism (PE), remains a clinical challenge. Diagnosis traditionally combines D-dimer assay and imaging guided by pretest clinical probability [1]. The latter involves the assessment of the likelihood of having VTE before any additional testing using validated scores based on clinical and paraclinical elements [2]. A fixed D-dimer threshold of 500 μg/L is usually used to rule out VTE when results are negative due to its validated high negative predictive value (NPV), while a positive result requires imaging because of a low specificity [1].

D-dimers are a fibrin degradation product. They rise in conditions involving increased fibrin generation such as VTE, inflammation contexts, trauma, hematomas, postoperative states, infections, malignancies, vascular anomalies, and coagulation disorders [3]. Physiological increases also occur with age, pregnancy, and postpartum, reflecting hypercoagulability [3,4]. Elevated D-dimer levels, although sensitive, lack specificity for acute VTE. This complicates the interpretation of elevated D-dimer especially in older populations who have higher baseline levels of D-dimer and often lead to unnecessary imaging [5,6].

To address this, multiple studies have tried to adjust D-dimer threshold to age [4,[7], [8], [9]]. Currently, the most widely used formula multiplies age by 10 for patients over 50 years old [7].

In this review, the validation of the age-adjusted D-dimer threshold will be discussed, as well as the strengths and limitations of the different D-dimer assay techniques when using the age-adjusted D-dimer threshold, which may influence results and clinical decisions [3].

## 2 The Age-Adjusted D-Dimer Threshold

While D-dimer sensitivity for PE exclusion was excellent at 100% (95% CI, 98%-100%; VIDAS and STA-Liatest) across all age groups suspected of PE with the conventional fixed 500 μg/L D-dimer threshold, specificity declined markedly with age because of a significant age-related rise in D-dimer levels [10]. Righini et al. [11] reported that for patients with suspected PE over 80 years, D-dimer excluded PE in only 5% (95% CI, 2%-10%) compared with 58% (95% CI, 51%-65%) in those under 40 years. Interpretation is therefore complicated in older populations where PE incidence among those suspected of PE increases from 12% in patients under 40 years to 44% in patients over 80 years [11,12].

To address this limitation, Righini et al. [4] proposed an age-adjusted D-dimer threshold in 2001. A retrospective analysis of 1029 patients with suspected PE suggested that a threshold of 900 μg/L improved specificity in patients over 70 years while maintaining a sensitivity of 95% and an NPV of 91%. Subsequent studies have explored thresholds ranging from 600 to 1000 μg/L, but consensus remains elusive [13]. For instance, a retrospective cohort using the VIDAS assay reported a 1000 μg/L threshold achieving over 98% sensitivity in patients over 60 years [14]. Specificity improved significantly, reaching 55% for patients aged 60 to 80 years (compared with 25.3% at the standard threshold) and 27% for those over 80 years (compared with 5% at 500 μg/L) [14]. Similarly, Haas et al. [15] proposed a 750-μg/L threshold for DVT exclusion in patients over 60 years, showing enhanced specificity without sacrificing sensitivity when using STA-Liatest, Tina-quant, and Innovance assays. However, the study’s lower NPV 95% CI decreased to <90%, failing to meet the Clinical and Laboratory Standards Institute and the Food and Drug Administration safety standards [16]. Both recommend a lower NPV 95% CI over 95% [16].

Despite improvements in specificity, Righini et al. [4] strongly discouraged thresholds >500 μg/L in patients over 60 years due to high false-negative rates. While alternative strategies, such as adding 100 μg/L per decade over age 60 years [15,17] or inversely calculating thresholds based on age (eg, 500 ng/mL plus 10 times the difference between 66 and the patient’s age for those under 66 years) [18] or multiplying age by a fixed factor (eg, 16 for those over 70 years) [19] have been proposed, none have demonstrated sufficient safety for widespread clinical adoption.

The 2010 study by Douma et al. [20] marked a significant advancement in the development of the age-adjusted D-dimer threshold. This post hoc analysis of 4 retrospective, multicenter studies, included over 5000 patients with suspected PE and nonhigh clinical probability who underwent D-dimer testing using VIDAS and Tina-quant assays across Belgium, France, Netherlands, and Switzerland. Patients were followed up for 3 months, and receiver operating characteristic curves were constructed for 10-year age groups beginning at age 50 years to determine optimal thresholds. The analysis revealed an 11.2 μg/L increase in the optimal threshold per decade starting at age 50 years, leading to the following formula: age × 10 μg/L (eg, 730 μg/L for a 73-year-old patient). Applying this age-adjusted threshold in patients over 50 years increased the proportion of negative D-dimer results by 11% to 18%, depending on the cohort, reducing the need for imaging. Diagnostic safety remained uncompromised, with a false-negative rate of <1% across all groups. Overall, up to 25% to 30% of patients over 50 years had PE safely excluded using this method. Despite its strengths, the study had limitations. Validation was restricted to retrospective analysis and 2 D-dimer assays. Although no significant differences were observed between these techniques, interassay variability highlights the need for validation across other testing platforms. This study not only derived the age-adjusted threshold but also demonstrated its potential to improve diagnostic efficiency and reduce imaging dependency in clinical practice.

## 3 Diagnostic Value of the Age-Adjusted D-Dimer Threshold Multiplying the Patient’s Age by 10

### 3.1 Efficiency and safety of the age-adjusted threshold in VTE diagnosis: a 10-year overview

**Figure 1:**
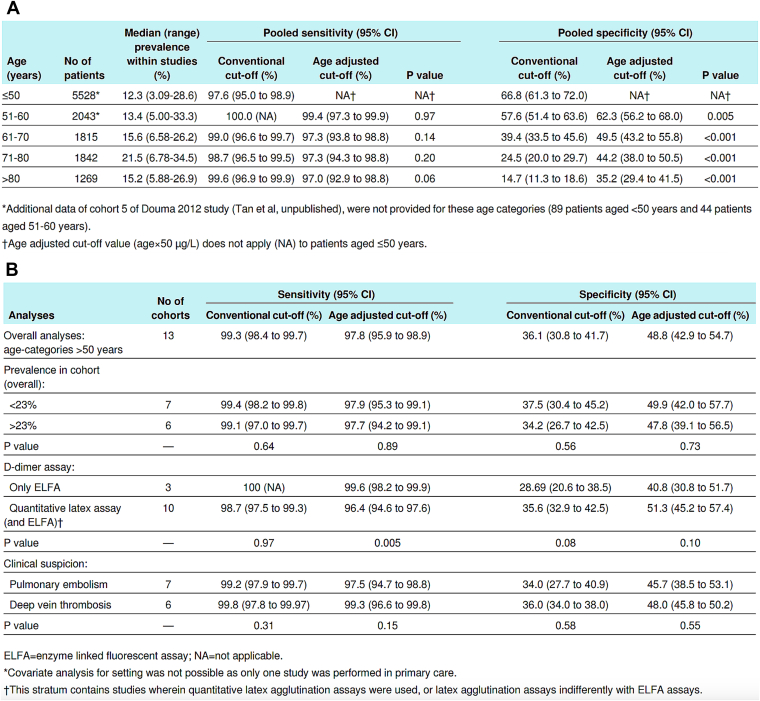
Diagnostic performance of D-dimers in patients over 50 suspected of VTE with nonhigh clinical probability, with or without the use of the age-adjusted threshold (A) according to various factors in patients with suspected VTE and a nonhigh clinical probability (B) [21].

While Douma et al. [20] derived the age-adjusted D-dimer formula, its validation in terms of efficiency and safety was achieved over the following years. Efficiency was defined as the proportion of patients in whom imaging could be safely withheld based on a nonhigh pretest clinical probability combined with a negative D-dimer result, whereas safety corresponded to the 3-month incidence of symptomatic VTE (failure rate). Both parameters were evaluated in several validation studies. A 2013 systematic review and meta-analysis by Schouten et al. [21] encompassing 13 large cohorts (12,497 patients) demonstrated that specificity declines significantly with age when the conventional threshold is used. The specificity went from 66.8% (95% CI, 61.3%-72%) in patients under 50 years to 14.7% (95% CI, 11.3%-18.6%) in those over 80 years (Figure 1A). The use of the age-adjusted threshold for patients over 50 years doubled significantly specificity in patients over 80 years (35.2%; 95% CI, 29.4%-41.5%) while maintaining a high sensitivity (>97%) across all age groups. It increases the proportion of patients in whom VTE can be excluded from 12.4% (conventional threshold: 500 μg/L) to 30.3%, with sensitivity remaining >97%. For patients over 80 years, the positive predictive value (PPV) improves to 21.1% (95% CI, 19.1%-23.2%), approximating the PPV of 29.2% (95% CI, 25.3%-33.1%) observed in patients under 50 years using the conventional threshold. This adjustment restores efficacy in older populations to levels comparable with those in younger cohorts while maintaining a high sensitivity. Subgroup analyses confirm no significant differences in diagnostic performances between PE and DVT, reinforcing the threshold’s applicability across VTE subtypes. A 2021 meta-analysis of over 40,000 patients further confirmed the reliability of this threshold for PE exclusion in patients with nonhigh clinical probability. However, variability in VTE incidence (12.3%-21.5%) across studies brings challenges for external validation [21].

**Figure 2:**
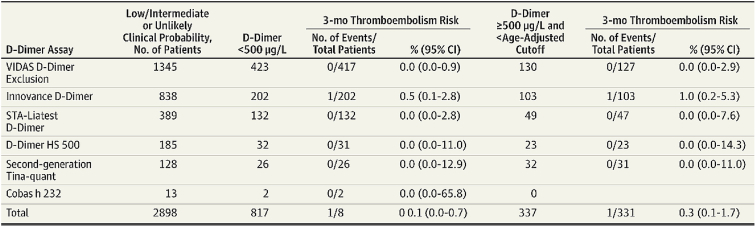
Results of the ADJUST-PE study according to D-dimer assays [7].

While retrospective analyses highlight its strong diagnostic performance, prospective validation remains essential to confirm its safety in clinical practice. The ADJUST-PE study [7], published in 2014, was the first prospective evaluation of the age-adjusted D-dimer threshold in PE diagnosis. Conducted across 19 emergency departments (EDs) in 4 European countries (Belgium, France, Netherlands, and Switzerland), the study included 3346 patients with suspected PE. Six validated D-dimer assays were used (VIDAS D-Dimer Exclusion [bioMérieux], second-generation Tina-quant [Roche], Cobas h 232 [Roche], STA-Liatest D-Dimer [Stago], HemosIL D-Dimer HS 500 [IL Diagnostics], and Innovance D-Dimer [Siemens]). A threshold of 500 μg/L was applied for patients under 50 years, and the age-adjusted formula (age × 10 μg/L) was used for those over 50 years. The primary endpoint was the incidence of VTE within 3 months following exclusion based on D-dimer testing. Among patients over 75 years, the age-adjusted threshold increased the proportion of negative D-dimer tests from 6.4% (95% CI, 4.8%-8.5%) to 29.7% (95% CI, 26.4%-33.3%), reducing CT imaging by one-third without increasing false-negative rates. The overall false-negative rate was 0.3% (95% CI, 0.1%-1.7%), within International Society of Thrombosis and Haemostasis (ISTH) safety margins. According to ISTH recommendations, the safety of any diagnostic strategy excluding VTE based on D-dimer levels should be established by ensuring that the upper limit of the 95% CI for the failure rate remains <3%, thereby defining the accepted safety margin. Results were consistent across all assays, with false-negative rates ranging from 0% to 1% (Figure 2). The findings confirmed the safety and efficiency of the age-adjusted threshold multiplying the patient’s age by 10 across various testing platforms, particularly in older populations, with a 5-fold increase in VTE exclusion significantly reducing imaging. This study established the age-adjusted threshold as a major criterion in the diagnostic strategy for PE, especially in patients over 75, ensuring both safety and efficiency in clinical practice.

The RELAX-PE study [8] published in 2021 also provided significant insights as the first prospective diagnostic strategy study since the ADJUST-PE study [7]. Conducted across Belgium, France, and Switzerland, 1507 outpatients with suspected PE and nonhigh clinical probability were included. Validated D-dimer assays (VIDAS, Innovance, STA-Liatest, AxSYM, HemosIL DD HS, and DPC Immulite 2000) were used. The study applied the age-adjusted thresholds (age × 10 μg/L) for patients over 50 years. The 3-month VTE rate was 0% (95% CI, 0%-1.41%) in patients over 50 years whose D-dimer levels fell between the conventional and the age-adjusted thresholds. Diagnostic efficiency improved significantly, with PE exclusion without imaging increasing by 20% overall, 35% in patients over 50 years, and 67% in those over 75 years. These findings align with the ADJUST-PE study [7]. The 2 studies combined showed an overall 3-month VTE risk of 0.17% (95% CI, 0.03-0.94), reinforcing the safety and efficacy of the age-adjusted threshold [7,8]. Its application has no upper age limit, making it especially valuable for older populations with naturally elevated D-dimer levels [7,8]. The strategy has been endorsed by multiple scientific societies [1] thought one of the biggest challenge remains assay variability near the exclusion threshold. The Clinical and Laboratory Standards Institute recommends a coefficient of variation of <7.5% for reliable D-dimer measurement [16]. Prospective diagnostic strategy studies address this concern by confirming the safety of the approach through the 3-month follow-up.

**Figure 3:**
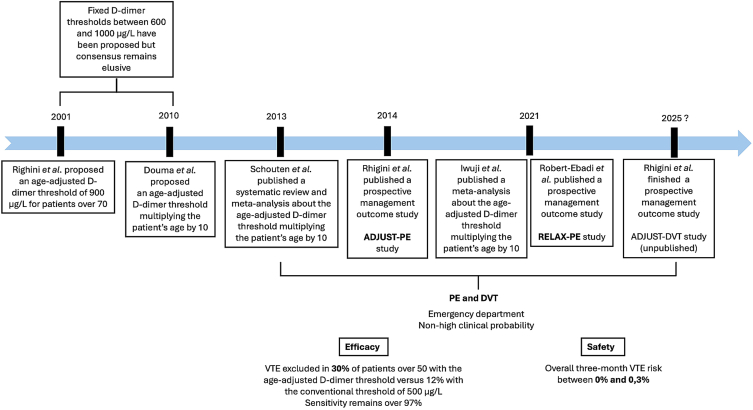
Historical milestones in the validation of the age-adjusted D-dimer threshold.

Overall, the age-adjusted D-dimer threshold demonstrated enhanced specificity without compromising sensitivity in excluding VTE, particularly in patients with suspected PE and nonhigh clinical probability in ED settings (Figure 3) [7,8,20]. However, real-world diagnostic strategy studies remain limited particularly for DVT [7,8]. Retrospective studies dominate this area, and the cautious stance of scientific societies reflects the absence of large-scale prospective trials [7,8]. Emerging evidence suggests the age-adjusted threshold may be as safe and efficient as the conventional approach for DVT exclusion. Some studies reported a higher sensitivity of the age-adjusted threshold for DVT than for PE [[22], [23], [24]]. However, a prospective diagnostic strategy study, the ADJUST-DVT study [25], follows the ADJUST-PE’s methodology to evaluate the 3-month incidence of DVT. The study is unpublished yet, but the abstract is available. Conducted in several centers in Belgium, Canada, Switzerland, and France, 3205 patients were included. The 3-month failure rate in patients with a D-dimer of >500 μg/L, but below the age-adjusted threshold was 0% (95% CI, 0%-2.3%). Overall failure rate of the strategy was 0.7% (95% CI, 0.4%-1.2%) [25].

### 3.2 The impact of D-dimer testing techniques on the age-adjusted threshold multiplying the patient’s age by 10

D-dimer in whole blood or plasma is measured using monoclonal antibodies that specifically recognize an epitope unique to cross-linked D-dimer [5]. More than 30 commercial assays are available for D-dimer testing, but they can be broadly categorized into 3 types of methods: whole-blood agglutination assays, enzyme-linked immunosorbent or enzyme-linked immunofluorescent assay (ELFA), and latex agglutination assays (immunoturbidimetric methods) [3]. Among these, latex agglutination assays are particularly favored because of their compatibility with automated coagulation analyzers allowing for fast and reliable plasma D-dimer measurement [3]. The European Society of Cardiology (ESC) recommends the use of the age-adjusted D-dimer threshold (age × 10 μg/L) exclusively for ELFA and immunoturbidimetric techniques [1]. Results obtained from D-dimer assays are not directly comparable, even when similar analytical methods are used, as significant variability exists between assays. Data from 2014 College of American Pathologists external quality program demonstrated intramethod coefficients of variation ranging from 6.4% to 17.7% and intermethod coefficients of variation ranging from 24% to 42% [26]. The absence of internationally recognized reference measurement procedures undermines the accuracy and reliability of D-dimer testing, potentially leading to misdiagnosis or underdiagnosis and limiting its broader clinical utility [27]. For instance, 1 study reported D-dimer levels for an 86-year-old patient with PE ranging from 590 to 1170 μg/L across 5 assays, potentially leading to divergent clinical decisions [28]. This lack of standardization arises from differences in the monoclonal antibodies used and variations in assay protocols and instrumentation [3,29]. The Fibrinolysis and DIC Standardization Subcommittees of the ISTH have therefore called for greater harmonization [30]. The diversity of fibrin degradation fragments produced by plasmin digestion and the differing specificities of monoclonal antibodies targeting D-dimer epitopes largely account for this lack of harmonization [3]. The age-adjusted thresholds enhances specificity but is influenced by variability in assay performance and patient characteristics complicating D-dimer’s interpretation [3]. This underscores the critical need for assay-specific validation of the age-adjusted threshold before it can be implemented into routine clinical practice. The impact of assay variability has been extensively studied [7,[31], [32], [33]]. There is a degree of variability between the 2 recommended analytical methods for D-dimer measurement [1]. Schouten et al. [21] reported superior diagnostic performance of the age-adjusted threshold when applied exclusively with the ELFA method, compared with combined use of immunoturbidimetric and ELFA assays, achieving a sensitivity of 99.6% (Figure 1B). These results are consistent with the VIDAS assay (an ELFA-based method) being the most extensively validated D-dimer assay for both the conventional and age-adjusted thresholds [7,[31], [32], [33]]. Furthermore, the DiET-PE study reanalyzed data from the original DiET study, which validated the STA-Liatest assay, an immunoturbidimetric method, for Food and Drug Administration approval using the conventional D-dimer threshold, demonstrating an NPV of 99.7% (95% CI, 99%-100%) [34].

When the age-adjusted D-dimer threshold was applied, the NPV remained high at 99.5% (95% CI, 98.7%-99.9%), but sensitivity declined slightly from 97.6% (95% CI, 91.7%-99.7%) to 95.2% (95% CI, 88.3%-98.7%). Only 1.9% of additional patients were excluded using the age-adjusted threshold, compared with 11.6% using the conventional D-dimer threshold. This limited gain may be explained by the exclusion of patients over 80 years in the DiET study [34]. Robert-Ebadi et al. [35] later performed a secondary analysis of the ADJUST-PE study, evaluating various D-dimer assays with the age-adjusted threshold to exclude PE. The results showed that the probability of reclassification from a positive result on the VIDAS assay to a negative result using the STA-Liatest assay was 19.6% (95% CI, 15.5%-24.3%) [35]. Therefore, the use of the age-adjusted D-dimer threshold with the STA-Liatest assay is not recommended [36].

**Figure 4:**
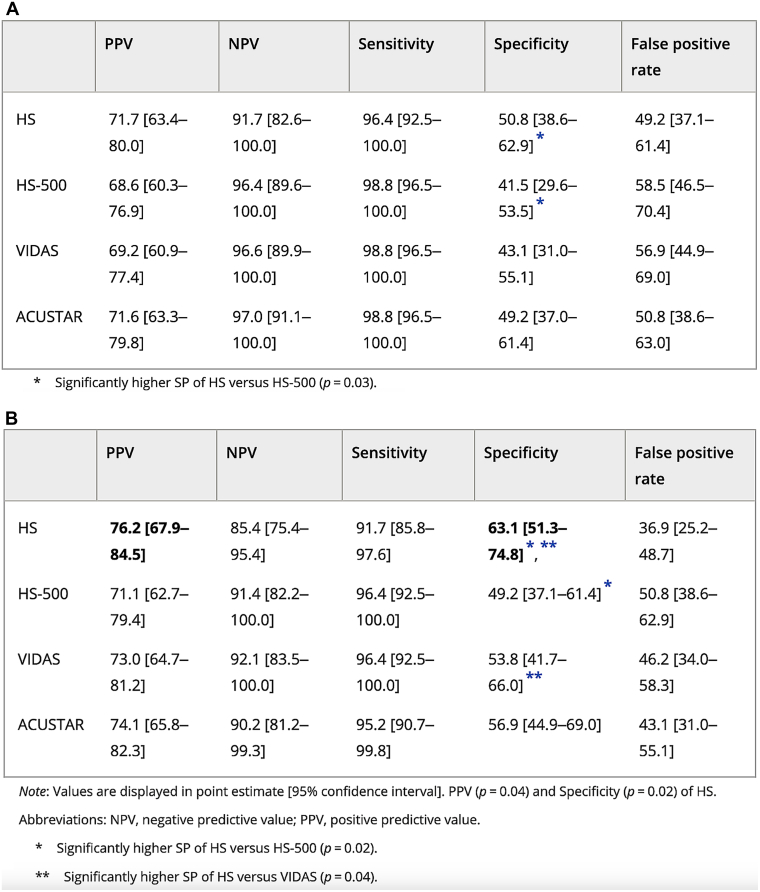
Diagnostic performance of different D-dimer assays using the manufacturer’s threshold (A) and the age-adjusted threshold (B) [38]. In bold, the results that are significantly different from the manufacturer’s threshold.

Variability was observed within the same analytical method and even between reagents from the same manufacturer. Both STA-Liatest and HemosIL D-Dimer assays are immunoturbidimetric methods. However, the former was not validated for use with the age-adjusted threshold, whereas the latter was [36,37]. Furthermore, a multicenter study across 7 EDs reported significantly lower specificity using HemosIL D-Dimer HS500 reagent than that using the HemosIL D-Dimer HS reagent, both with and without application of the age-adjusted threshold resulting in increased false-positive results and unnecessary imaging (Figure 4A, B) [38]. Taken together, these results highlight that variability in assay and reagent performance must be carefully considered when implementing the age-adjusted D-dimer threshold to ensure diagnostic accuracy and maintain patient safety.

### 3.3 Efficiency and safety of the HemosIL D-dimer HS 500 reagent for the age-adjusted threshold

**Figure 5:**
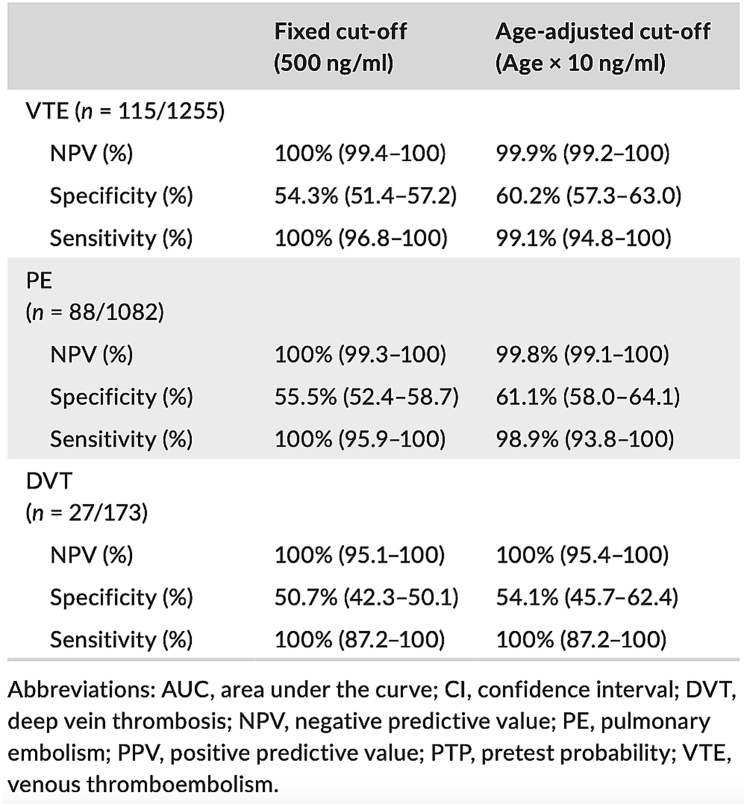
Performance of the HemosIL D-dimer HS 500 test in diagnosing VTE using the fixed conventional threshold (500 μg/L) and age-adjusted threshold (age × 10) [37].

While the age-adjusted D-dimer threshold has been extensively validated across various D-dimer assays, data specific to the HemosIL D-dimer HS 500 reagent remains limited compared to others in the HemosIL range [7,21,28,[38], [39], [40], [41], [42], [43]]. Existing evidence suggests that the HemosIL D-dimer HS 500 reagent offers comparable diagnostic performance to the HemosIL D-dimer HS reagent using both conventional and age-adjusted thresholds despite being described as less sensitive and significantly more specific [38]. These differences are not expected to compromise the clinical utility of the age-adjusted threshold. The evaluation of the HemosIL D-dimer HS-500 reagent using the age-adjusted threshold is mostly based on retrospective studies including approximately 6000 patients (Table 1) [7,23,37,41,[44], [45], [46], [47], [48], [49]]. These studies conducted primarily in ED across several European countries reported VTE incidence between 3.6% and 20.7%, which is consistent with the estimated incidence for a nonhigh clinical probability of VTE [50]. Diagnostic performance has been consistently strong, with NPVs ranging from 95% to 100%. For the HemosIL D-dimer HS reagent, which reports results in D-dimer units (DDUs), the conventional threshold is 250 μg/L, equivalent to 500 μg/L fibrinogen equivalent units (FEUs). Therefore, when using the age-adjusted threshold in DDUs, the patient’s age is multiplied by 5 instead of 10. Several studies [23,47] converted results to FEU to adjust the threshold. The age-adjusted D-dimer threshold enhanced specificity while maintaining a high sensitivity with the HemosIL D-dimer HS 500 reagent. De Pooter et al. [37] included in a multicenter study 1255 patients with suspected VTE and evaluated the HemosIL D-dimer HS 500 reagent. Compared with the conventional threshold, the age-adjusted threshold improved specificity from 54.3% (95% CI, 51.4%-57.2%) to 60.2% (95% CI, 57.3%-63%), while sensitivity remained > 99% (99.1%; 95% CI, 94.8%-100%) (Figure 5). This adjustment resulted in a 5.9% absolute increase in the proportion of patients for whom PE could be excluded without imaging, consistent with literature report of a 5% absolute increase [51]. The study reported an NPV of 99.9% (95% CI, 99.2%-100) (Figure 5). Despite its retrospective design, the study provided evidence for the reagent’s clinical utility. Another limitation is the underrepresentation of patients with suspected DVT (173) compared with those with PE (1082) [37]. This disparity may be explained by the limited sensitivity of D-dimer testing for distal DVT leading to the frequent use of lower-limb venous ultrasound in EDs rather than D-dimer testing [52]. An underuse rate of 84.4% for D-dimer testing in DVT assessments has been reported, which is among the highest rates in clinical diagnostics [53]. In the ADJUST-PE study, the age-adjusted threshold using HemosIL D-dimer HS-500 reagent yielded a zero VTE rate over 3 months of follow-up though the upper CI exceeded 3% (Figure 2) [7]. However, this reagent was not included in the RELAX-PE study [8]. Gómez-Jabalera et al. [47] assessed different age-multiplying factors for threshold adjustment using HemosIL D-dimer HS 500 reagent. Their findings demonstrated optimal diagnostic performance with a multiplier of 10 in patients with intermediate pretest clinical probability (NPV, 100%; 95% CI, 72.2%-100%; specificity, 50%; 95% CI, 29.9%-70.1%) and a multiplier of 25 in patients with low pretest clinical probability (NPV, 100%; 95% CI, 94.8%-100%; specificity, 76.1%; 95% CI, 66.4%-83.6%). These results validated its use for VTE exclusion in clinical practice.

**Table 1:** Key studies evaluating the diagnostic performance of the age-adjusted threshold with HemosIL D-dimer reagent.

Year of publication	Study	Threshold	Design	Country	Age (y), mean or median	No. of patients whose D-dimers were measured with HemosIL Reagent	Reagent	VTE	VTE incidence (%)	Clinical probability score	Diagnostic performance of the age-adjusted threshold (%) (95% CI)	Validation of the use of the age × 10 threshold
2014	Cini et al. [48]	200-230 μg/L <50 y Age × 5 > 50 y 376 μg/L >60 y	Prospective	Italy	67 (mean)	326	HemosIL D-dimer HS (DDU)	DVT	11.1	Wells score	Sensitivity, 100 (88.8-100) NPV, 100 (97.8-100) Specificity, 67.3 (61.2-73.2) PPV, 27.7 (19.7-36.9)	Yes
2014	Righini et al. [7]	500 μg/L <50 y Age × 10 > 50 y	Diagnostic strategy study	Switzerland, Belgium, Holland, France	63 (median)	185	HemosIL D-Dimer HS 500 (FEU)	PE	19	Wells score or revised Geneva score	No information	Yes
2018	Jaconelli et al. [41]	250 μg/L <50 y Age × 5 > 50 y	Retrospective	England	54 (median)	1649 (986 PE and 663 DVT)	HemosIL D-dimer HS (DDU)	PE/DVT	3.6 (PE)	Wells score	Sensitivity. 95 (86.1-99.0) NPV, 99.7 (99.1-99.9) Specificity, 78 (75.6-80.3) PPV, 17 (13.2-21.5)	Yes
2017	Nobes et al. [46]	250 μg/L <50 y Age × 5 > 50 y	Retrospective	Scotland	68 (mean)	1000	HemosIL D-dimer HS (DDU)	PE	10.1 and 20.7	Revised Geneva score	Sensitivity, 97.5 (94.7-99. 1) NPV, 96.9 (93.1-98.7) Specificity, 24.9 (21.8-28.1)	Yes
2018	Lim et al. [49]	200-230 μg/L <50 y Age × 5 > 50 y 376 μg/L >60 y	Retrospective	Australia	58.5 (mean)	176	HemosIL D-dimer HS (DDU)	PE	17	Wells score	NPV, 95 (85-99) PPV, 18 (11-28)	Yes
2018	Gomez et al. [47]	500 μg/L Age × 10 μg/L Age × 15 μg/L Age × 20 μg/L Age × 25 μg/L Age × 30 μg/L	Retrospective	Spain	71.6 (mean)	138	HemosIL D-Dimer HS 500 (FEU)	DVT	16.7	Wells score	Sensitivity, 100 (70.1-100) NPV, 100 (72.2-100) Specificity, 50 (29.9-70.1) PPV, 47.4 (27.3-68.3)	Yes for patients with intermediate clinical probability
2020	Jimenez et al. [45]	250 μg/L <50 y Age × 5 > 50 y Converted to FEU so 500 μg/L <50 y Age × 10 > 50 y	Nonrandomized prospective	Spain	69.3 (mean)	606	HemosIL D-dimer HS (DDU)	DVT	10	Wells score	Sensitivity, 76 (0.60-0.88) NPV, 97 (0.95-0.99) Specificity, 61 (0.57-0.65) PPV, 12 (0.09-0.17)	No
2021	De Pooter et al. [37]	500 μg/L <50 y Age × 10 > 50 y	Retrospective analysis on data from a multicenter prospective study	France	59 (median)	1255 (1082 PE and 173 DVT)	HemosIL D-dimer HS 500 (FEU)	PE/DVT	13.7	Wells score	Sensitivity, 99.1 (94.8-100) NPV, 99.9 (99.2-100) Specificity, 60.2 (57.3-63)	Yes
2022	Barrett et al. [23]	230 μg/L <50 y Age × 5 > 50 y Converted to FEU so 500 μg/L <50 y Age × 10 > 50 y	Retrospective	England	No information	1236 (394 PE and 842 DVT)	HemosIL D-dimer HS (DDU)	PE/DVT	18.28	Wells score	Sensitivity, 98.67 (96.17-99.73) NPV, 99.00 (97.13-99.69) Specificity, 31.09 (28.24-34.05) PPV, 24.4 (23.46-25.09)	Yes

DDU, D-dimer unit; DVT, deep vein thrombosis; FEU, fibrinogen equivalent unit; NPV, negative predictive value; PE, pulmonary embolism; PPV, positive predictive value.

While most studies support the validation of the age-adjusted threshold for VTE exclusion using the HemosIL D-dimer reagent (Table 1) [7,23,37,41,[44], [45], [46], [47], [48], [49]] some studies remained cautious. Jimenez-Guiu et al. [45] reported a higher false-negative rate of 24% with the age-adjusted threshold that that of 7% with the conventional threshold for DVT exclusion. This result was attributed to the use of complete lower-limb venous ultrasound, which may have detected distal DVT cases with lower D-dimer levels increasing false-negative rates [54]. Other studies report false-negative rates ranging between 0% and 4.8% [23,37,41,44,[46], [47], [48]]. Despite these challenges, the age-adjusted D-dimer threshold for the HemosIL D-dimer reagent has demonstrated not only diagnostic accuracy but also economic benefits. By reducing imaging needs, it is estimated to lower diagnostic costs by 6.7% [37]. When applied with the HemosIL D-dimer reagent, the age-adjusted threshold represents a valuable tool in clinical practice, due to its improved specificity, high NPV, and cost-effectiveness (Table 2) [55,56].

**Table 2:** Key studies evaluating the diagnostic performance of the age-adjusted threshold multiplying the patient’s age by 10.

Year of publication	Study	Country	Design	VTE	No. of included patients (% male)	No. of patients with nonhigh clinical probability of VTE	Mean age (y)	Incidence of VTE over the included patients (%)	Setting	D-dimer cutoff	CDR used (cutoff for nonhigh clinical probability)	D-dimer assay	Diagnostic performances (%) (95% CI)	Efficiency	Validation of the age-adjusted threshold
1999	Le Blanche et al. [17]	France	Prospective cohort study	DVT	150 (37.5)	NA	86.3	35.3	Geriatric inpatients	(a) 500 μg/L over 70 y old (b) 750 μg/L over 70 y old	NA	VIDAS D-dimer	(a) Sensitivity, 98.1 (94.3-100) NPV, 83.3 (53.4-100) (b) Sensitivity, 98.1 (94.3-100) NPV, 95 (85.4-100)	Exclusion of VTE in 12.7% of cases with the 750-μg/L threshold and 3.3% of cases with the 500 μg/L threshold	Yes
2000	Righini et al. [11]	Switzerland and Canada	Prospective cohort study	PE	1029	931	Unspecified	27	Emergency department	500 μg/L for all ages	Clinical intuition (<80%)	STAGO Asserachrom D-dimer enzyme immunoassay VIDAS D-dimer	Sensitivity, 99.6 (98-100) Specificity, 47 (44-51)	Exclusion of PE in 58% of patients under 40 y old and 5% of patients over 80 y old	NA
2007	Harper et al. [14]	New Zealand	Retrospective cohort study	PE and DVT	1897	Unspecified	Unspecified	Unspecified	Emergency department	(a) 500 μg/L for 60-80 y old (b) 750 μg/L for 60-80 y old (c) 1000 μg/L for 60-80 y old	Wells score (<2 for DVT; <5 for PE)	VIDAS D-dimer	Sensitivity for all thresholds, 100 (a) Specificity, 25.3 (b) Specificity, 43.4 (c) Specificity, 55	NA	Yes
2009	Haas et al. [15]	Not given	Retrospective cohort study	DVT	466	Unspecified	60	39	Outpatient clinic	Low pretest probability value and 750 μg/L over 60 y old	Wells score (<2)	STA-Liatest Tina-quant Innovance	STA-Liatest Sensitivity, 100 (87.2) Specificity, 48.5 (36.3) NPV, 100 (86.4) Tina-quant Sensitivity, 100 (88.8) Specificity, 60.6 (42.8) NPV, 100 (83.8) Innovance Sensitivity, 100 (91.8) Specificity, 49.2 (32.1) NPV, 100 (89) The 95 lower level of confidence are given in parenthesis.	NA	Yes
2010	Douma et al. [20], derivation set	Belgium, France, Netherlands, and Switzerland	Retrospective cohort study	PE	1721 (41)	1331	61	24	Emergency department or outpatient clinic	(a) D-dimer <500 μg/L for all ages (b) D-dimer <age × 10 over 50 y old	Wells (<5)	VIDAS D-dimer	NA	Exclusion of VTE in 42% of cases with the age × 10 threshold vs 36% of cases with the 500-μg/L threshold Failure rate (all ages), 0.2% (95% CI, 0%-1%)	Yes
2010	Douma et al. [20], validation set 2	Belgium, France, Netherlands, and Switzerland	Retrospective cohort study	PE	1819 (49)	1643	59	21	Emergency department or outpatient clinic	(a) D-dimer <500 μg/L for all ages (b) D-dimer <age × 10 over 50 y old	Revised Geneva score (<10)	VIDAS D-dimer	NA	Exclusion of VTE in 40% of cases with the age × 10 threshold vs 34% of cases with the 500-ng/mL threshold Failure rate (all ages), 0.3% (95% CI, 0.1%-1.1R)	Yes
2010	Douma et al. [20], validation set 1	Belgium, France, Netherlands, and Switzerland	Retrospective cohort study	PE	3306 (43)	2158	53	20	Emergency department or outpatient clinic	(a) D-dimer <500 μg/L for all ages (b) D-dimer <age × 10 over 50 y old	Wells (<5)	VIDAS D-dimer Tina-quant	NA	Exclusion of VTE in 50% of cases with the age × 10 threshold vs 45% of cases with the 500-ng/mL threshold Failure rate (all ages), 0.6% (95% CI, 0.3%-1.3%)	Yes
2012	Douma et al. [40], cohort 1	Netherlands	Retrospective cohort study	DVT	812 (36)	472	59	39	Emergency department or outpatient clinic	(a) D-dimer <500 μg/L for all ages (b) D-dimer <age × 10 over 50 y old	Wells (<2)	Tina-quant	NA	Exclusion of DVT in 41% of cases with the age × 10 threshold vs 36% of cases with the 500-μg/L threshold Failure rate (all ages) with the age × 10 threshold, 1% (95% CI, 0.3%-3.7%)	Yes
2012	Douma et al. [40], cohort 2	Switzerland and Canada	Retrospective cohort study	DVT	474 (38)	419	61	23	Emergency department or outpatient clinic	(a) D-dimer <500 μg/L for all ages (b) D-dimer <age × 10 over 50 y old	Clinical probability estimated by treating doctor (<80%)	VIDAS D-dimer	NA	Exclusion of DVT in 39% of cases with the age × 10 threshold vs 30% of cases with the 500-μg/L threshold Failure rate (all ages) with the age × 10 threshold, 1.2% (95% CI, 0.2%-4.3%)	Yes
2012	Douma et al. [40], cohort 3	Italy, Canada, France, and South Africa	Retrospective cohort study	DVT	359 (41)	297	66	23	Emergency department or outpatient clinic	(a) D-dimer <500 μg/L for all ages (b) D-dimer <age × 10 over 50 y old	Wells (<2)	STA-Liatest	NA	Exclusion of DVT in 53% of cases with the age × 10 threshold vs 43% of cases with the 500 μg/L threshold Failure rate (all ages) with the age × 10 threshold, 1.3% (95% CI, 0.2%-4.6%)	Yes
2012	Douma et al. [40], cohort 4	Canada	Retrospective cohort study	DVT	556 (38)	484	65	10	Emergency department or outpatient clinic	(a) D-dimer <500 μg/L for all ages (b) D-dimer <age × 10 over 50 y old	Wells (<2)	MDA D-dimer	NA	Exclusion of DVT in 69% of cases with the age × 10 threshold vs 59% of cases with the 500-μg/L threshold Failure rate (all ages) with the age × 10 threshold, 0.3% (95% CI, 0.1%-1.7%)	Yes
2012	Douma et al. [40], cohort 5	Netherlands	Retrospective cohort study	DVT	617 (52)	212	58	37	Emergency department or outpatient clinic	(a) D-dimer <500 μg/L for all ages (b) D-dimer <age × 10 over 50 y old	Wells (<2)	STA-Liatest Tina-quant	NA	Exclusion of DVT in 47% of cases with the age × 10 threshold vs 39% of cases with the 500-μg/L threshold Failure rate (all ages) with the age × 10 threshold, 0% (95% CI, 0%-3%)	Yes
2012	Douma et al. [40], all cohorts	Netherlands, Canada, Switzerland, France, South Africa, and Italy	Retrospective cohort study	DVT	2818	1884	61.8	26.4	Emergency department or outpatient clinic	(a) D-dimer <500 μg/L for all ages (b) D-dimer <age × 10 over 50 y old	Clinical probability estimated by treating doctor (<80%) Wells (<2)	STA-Liatest Tina-quant VIDAS D-dimer MDA D-dimer	NA	Failure rate (all ages) with the age × 10 threshold, 0.7% (95% CI, 0.4%-1.5%)	Yes
2013	Penaloza et al. [2]	USA, France, and Belgium	Retrospective cohort study	PE	4537	Unspecified	53	10.1	Emergency department or outpatient clinic	(a) D-dimer <500 μg/L for all ages (b) D-dimer <age × 10 over 50 y old	Revised Geneva score (<10)	STA-Liatest VIDAS D-dimer MDA D-dimer	(a) AUC of ROC curves, 0.901 (95% CI, 0.888-0.915) (b) AUC of ROC curves 0.893 (95% CI, 0.879-0.908)	Exclusion of PE in 46.3% of cases with the age × 10 threshold vs 36.1% of cases with the 500-μg/L threshold Failure rate (all ages) with the age × 10 threshold, 0.6% (95% CI, 0.3%-1%)	Yes
2013	Schouten et al. [21]	Belgium, France, Netherlands, Switzerland, Canada, South Africa, Italy, and USA	Systematic review and meta-analysis including 5 retrospective studies	PE and DVT	22,608	12,630	Unspecified	12.3-21.9; median (range) incidence between studies on patients with nonhigh clinical probability	Inpatients and outpatients	(a) D-dimer <500 μg/L for all ages (b) D-dimer <age × 10 over 50 y old	Wells (<2 for DVT <5 for PE) Revised Geneva score (<10) Clinical probability estimated by treating doctor (<80%)	VIDAS D-dimer Tina-quant STA-Liatest Innovance D-dimer HS MDA D-dimer	(a) <50 y old Sensitivity, 97.6 (95-98.9) Specificity, 66.8 (61.3-72) >50 y old Sensitivity, 99.3 (98.4-99.7) Specificity, 36.1 (30.8-41.7) (b) Sensitivity, 97.8 (95.9-98.9) Specificity, 48.8 (42.9-54.7)	Unspecified	Yes
2014	Righini et al. [55]	Belgium, France, Netherlands, and Switzerland	Prospective management outcome study	PE	3346	2898	66	19	Emergency department	(a) D-dimer <500 μg/L for all ages (b) D-dimer <age × 10 over 50 y old	Revised Geneva score (<5) Wells score (<5)	VIDAS D-dimer Innovance STA-Liatest HemosIL HS-500 Second-generation Tina-quant Cobas h-232	NA	Exclusion of VTE in 29.7% of cases with the age-adjusted threshold in individuals over 50 y old Failure rate (all ages) with the age × 10 threshold, 0.3% (95% CI, 0.1%-1.17%)	Yes
2016	van Es et al. [49]	Belgium, France, Netherlands, and Switzerland	Systematic review and meta-analysis including 6 prospective studies	PE	7268 (42)	5223	56	22	Inpatients and outpatients	(a) D-dimer <500 μg/L for all ages (b) D-dimer <age × 10 over 50 y old	Wells (<5)	VIDAS D-dimer Tina-quant STA-Liatest Innovance HemosIL HS	NA	Exclusion of PE in 33% of cases with the age × 10 threshold vs 28% of cases with the 500-μg/L threshold Failure rate (all ages) with the age × 10 threshold, 0.94% (95% CI, 0.58%-1.5%)	Yes
2017	Nybo et al. [22]	Belgium, France, Netherlands, Switzerland, Canada, South Africa, Italy, and USA	Systematic review including 3 prospective studies and 5 retrospective studies	DVT	10,772	Unspecified	Unspecified	4.2-51.6 (incidence between studies)	Inpatients and outpatients	(a) D-dimer <500 μg/L for all ages (b) D-dimer <age × 10 over 50 y old	Unspecified	VIDAS D-dimer Tina-quant STA-Liatest Innovance HemosIL HS MDA D-dimer AxSYM D-Dimer	(a) NPV, 89.7-100 (b) NPV, 91.8-100	NA	Yes
2021	Iwuji et al. [56]	Canada, UK, USA, Spain, Italy, and Europe (countries unspecified)	Systematic review and meta-analysis of 9 retrospective studies	PE	Unspecified	47,720	Unspecified	Unspecified	Unspecified	(a) D-dimer <500 μg/L for all ages (b) D-dimer <age × 10 over 50 y old	Unspecified	Unspecified (various D-dimer assays)	(a) Sensitivity, 98.8 (97.9-99.7) Specificity, 29.6 (11.4-47.7) (b) Sensitivity, 96 (93.8-98.2) Specificity, 41.3 (27-55.6)	NA	Yes
2021	Robert-Ebadi et al. [8]	Belgium, France, and Switzerland	Prospective management outcome study	PE	Unspecified	1507	Unspecified	Unspecified	Emergency department	(a) D-dimer <500 μg/L for all ages (b) D-dimer <age × 10 over 50 y old	Revised Geneva score (<4)	Unspecified (ELISA)	NA	20% increase in the proportion of negative D-dimer tests in the whole cohort with the age-adjusted threshold Failure rate (all ages) with the age × 10 threshold, 0% (95% CI, 0%-1.41%)	Yes
2022	Barrett et al. [23]	England	Retrospective cohort study	PE and DVT	1236	Unspecified	Unspecified	18.28	Emergency department	D-dimer <age × 10 Only patients over 50 y old were included	Wells score (<2 for DVT; <5 for PE)	HemosIL HS	Sensitivity, 98.67 (96.17-99.73) NPV, 99.00 (97.13-99.69) Specificity, 31.09 (28.24-34.05) PPV, 24.4 (23.46-25.09)	NA	Yes
2025	Righini et al. (unpublished)	Belgium, Canada, France, and Switzerland	Prospective management outcome study	DVT	3205	2169	Unavailable	14	Emergency department	(a) D-dimer <500 μg/L for all ages (b) D-dimer <age × 10 over 50 y old	Wells score (<2) Revised Geneva score (<4)	VIDAS D-dimer Innovance	NA	Failure rate (all ages) with the age × 10 threshold, 0.0%, (95% CI, 0.0%-2.3%)	Yes

AUC, area under the curve; DVT, deep vein thrombosis; NA, not applicable; NPV, negative predictive value; PE, pulmonary embolism; PPV, positive predictive value; ROC, receiver operating characteristic.

### 3.4 Medicoeconomic impact of the age-adjusted threshold

The diagnostic strategy for PE aims to reduce reliance on imaging such as computed tomography pulmonary angiography, thereby minimizing patient exposure to ionizing radiation and iodinated contrast agents, which carry risks like allergic reactions and contrast-induced nephropathy [57]. Safely excluding PE without imaging also shortens ED stays and lowers health care costs [58]. Annual expenditures for VTE management are estimated at €1.5 to €3.3 billion in Europe, and $7 to $10 billion in the United States [58,59]. Similar cost concerns apply to DVT where reliance on lower-limb venous ultrasound although radiation free increases ED workload and adds expenses. Adopting the age-adjusted D-dimer threshold demonstrated significant cost saving without compromising diagnostic accuracy [7,8,58]. Studies have projected annual savings exceeding $80 million in the United States with no impact on quality-adjusted life years and £26,529 in the United Kingdom [23,60,61]. A European study assessed imaging use before and after introducing the age-adjusted threshold in an ED [6]. Following a targeted intervention including a multidisciplinary meeting and a laboratory report promoting the age-adjusted D-dimer threshold, imaging rates dropped significantly. Computed tomography pulmonary angiography use declined from 14% to 5.3%, and venous ultrasound use rates reduced significantly from 22.5% to 12.9% [6]. These reductions translated into a 6.9% decrease in PE diagnostic costs and a 5.1% reduction for DVT amounting to €3080 and €3059 per 1000 suspected patients, respectively [37]. By improving specificity, reducing imaging reliance and lowering costs, the age-adjusted threshold offers a cost-effective strategy to VTE management.

### 3.5 Medical societies’ recommendation regarding the adoption of the age-adjusted threshold

**Figure 6:**
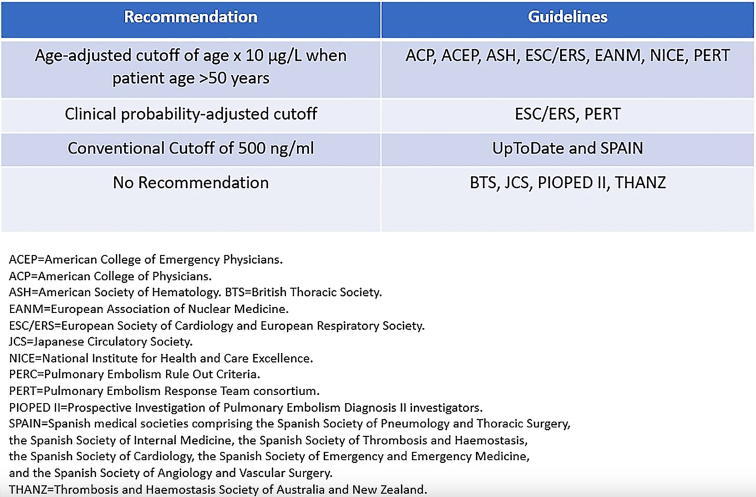
International recommendation on the age-adjusted D-dimer threshold for PE diagnosis [62].

The age-adjusted D-dimer threshold multiplying the patient’s D-dimer results by 10 in those over 50 years old gained widespread acceptance for patients with a nonhigh clinical probability of VTE. Several major medical societies including the ESC recommend its use either independently or within diagnostic strategies such as the YEARS algorithm (Figure 6) [1,62]. Recent studies validated the integration of the YEARS algorithm with the age-adjusted D-dimer threshold for PE diagnosis in terms of safety and efficiency [63]. A randomized trial comparing a combined approach (Pulmonary Embolism Rule-Out Criteria, YEARS algorithm, and age-adjusted threshold) to exclude PE with the conventional age-adjusted threshold reported noninferior outcomes achieving a 3-month PE incidence rate of 0.15% (95% CI, 0%-0.86%) vs 0.80% (95% CI, 0.26%-1.86%) [64]. These findings reinforce the clinical viability of combining diagnostic strategies to optimize patient outcomes [64]. However, adoption of the age-adjusted D-dimer threshold varies across guidelines [62]. Societies such as the National Institute for Health and Care Excellence, ESC, European Respiratory Society, European Association of Nuclear Medicine, Pulmonary Embolism Response Team, American College of Emergency Physicians, American Society of Hematology, and American College of Pathology explicitly recommend its use for patients over 50 years old for PE exclusion underlining its safety and efficiency [44,65]. However, some organizations including the Thrombosis and Hemostasis Society of Australia and New Zealand, the Japan Circulatory Society, and the British Thoracic Society, do not endorse the age-adjusted D-dimer threshold. Additionally, UpToDate guidelines recommend the age-adjusted threshold primarily for patients with a low clinical probability. This cautious stance is echoed in Spanish national guidelines, which favor the conventional threshold [65,66]. The ADJUST-PE study validated the efficiency and safety for PE diagnosis leading to a widespread endorsement of the age-adjusted D-dimer threshold for PE diagnosis [7,55]. However, its application to DVT remains less established [1]. While systematic reviews support its efficiency and safety for DVT, the absence of randomized trials has limited broader recommendations [22]. As evidence expands particularly with the ADJUST-DVT study [25], the adoption of the age-adjusted D-dimer threshold across VTE diagnostic pathways is expected to grow.

### 3.6 Toward an international implementation of the age-adjusted D-dimer threshold

The age-adjusted D-dimer threshold has gained validation across multiple continents through diverse methodologies and is supported by 2 diagnostic strategy studies [1,7,8,56]. These findings highlight its potential for widespread adoption, particularly in PE diagnosis. Recommendations from major scientific societies advocate for its implementation and the forthcoming results of the ADJUST-DVT study [25] may extend its role to DVT diagnosis [1,62,65]. Given the clinical overlap between PE and DVT, consensus on its broader application may soon emerge [67]. However, global implementation faces challenges. Variability in D-dimer assays stemming from differences in monoclonal antibodies, methods and clinical performance complicates the D-dimer standardization [3,68]. Diagnostic accuracy varies with VTE type and severity such as proximal vs distal DVT or subsegmental vs massive PE, requiring assays with high sensitivity and NPV to ensure a safe VTE exclusion [69]. Further complexity arises from inconsistent reporting units including nanograms per milliliter, micrograms per liter, FEUs, and DDUs, creating up to 28 potential combinations for age-adjusted threshold calculations [28,29,70]. Clear communication between laboratories and clinicians is relevant to ensure a correct D-dimer interpretation. The age-adjusted D-dimer threshold also faces competition from the YEARS algorithm, which adjusts the D-dimer threshold based on pretest clinical probability. Stals et al. [9] study suggests that the YEARS algorithm reduces imaging more efficiently leading to an increasing endorsement in clinical guidelines [1]. However, external validation studies have identified potential risks with the YEARS algorithm, including missed PE diagnoses when D-dimer levels reduce <1000 μg/L but exceed the age-adjusted D-dimer threshold (patient’s age × 10) [71]. Overall, an international implementation of the age-adjusted D-dimer threshold requires addressing assay variability, improving assay standardization, and enhancing clinician familiarity.

## 4 Conclusion

The age-adjusted D-dimer threshold is efficient and safe for patients over 50 with a nonhigh pretest probability of VTE. However, D-dimer results must be interpreted regarding the clinical context while considering assay variability and local diagnostic protocols. Global implementation requires adherence to manufacturer guidelines approved by regulatory authorities. Clear communication between laboratories and clinicians about D-dimer assay diagnostic performances is crucial for safe and efficient application to ensure optimal patient outcomes. Considering the literature findings and the reagent used, implementing an age-adjusted D-dimer threshold should begin by formalizing the application of the age-adjusted D-dimer threshold for VTE exclusion diagnosis in the laboratory report. Complementary educational initiatives, such as informational meetings, should also be undertaken to ensure clinicians are aware of methodological variability across assays and apply the age-adjusted threshold appropriately in this context.
